# Multidrug-Resistant *Shigella* Infections in Patients with Diarrhea, Cambodia, 2014–2015

**DOI:** 10.3201/eid2209.152058

**Published:** 2016-09

**Authors:** Kamonporn Poramathikul, Ladaporn Bodhidatta, Sivhour Chiek, Wilawan Oransathid, Sirigade Ruekit, Panida Nobthai, Woradee Lurchachaiwong, Oralak Serichantalergs, Chanthap Lon, Brett Swierczewski

**Affiliations:** Armed Forces Research Institute of Medical Sciences, Bangkok, Thailand (K. Poramathikul, L. Bodhidatta, W. Oransathid, S. Ruekit, P. Nobthai, W. Lurchachaiwong, O. Serichantalergs, B. Swierczewski);; Battambang Provincial Referral Hospital, Battambang, Cambodia (S. Chiek);; Armed Forces Research Institute of Medical Sciences, Phnom Penh, Cambodia (C. Lon)

**Keywords:** *Shigella*, antimicrobial resistance, Cambodia, dysentery, bacterial infections, bacteria, MDR genes, Cambodia, enteric infections, *Suggested citation for this article*: Poramathikul K, Bodhidatta L, Chiek S, Oransathid W, Ruekit S, Nobthai P, et al. Multidrug-resistant *Shigella* infections in patients with diarrhea, Cambodia. Emerg Infect Dis. 2016 Sep [*date cited*]. http://dx.doi.org/10.3201/eid2209.152058

## Abstract

We observed multidrug resistance in 10 (91%) of 11 *Shigella* isolates from a diarrheal surveillance study in Cambodia. One isolate was resistant to fluoroquinolones and cephalosporins and showed decreased susceptibility to azithromycin. We found mutations in *gyr*A, *par*C, β-lactamase, and *mph*A genes. Multidrug resistance increases concern about shigellosis treatment options.

Shigellosis is a major public health problem in developing countries. Antimicrobial therapy with fluoroquinolones is recommended to shorten the course of disease and fecal shedding. However, limitations on shigellosis treatment options have been a concern since 1993, when ciprofloxacin-resistant *Shigella* was documented (*1*), followed by reports of multidrug-resistant (MDR) *Shigella* and of *Shigella* that harbored extended-spectrum β-lactamase (ESBL) genes (*2*). We describe MDR *Shigella* isolated from patients with diarrhea in Cambodia during 2014–2015.

## The Study

During July 2014–April 2015, we examined stool specimens collected from patients 3 months–5 years of age and 18–60 years of age who were seen for or admitted with acute diarrhea at 3 healthcare settings in Battambang, Cambodia, as part of ongoing hospital-based surveillance of diarrhea etiology. Stool specimens were processed for identification of enteric pathogens by standard microbiology, ELISA, and PCR. *Shigella* species were identified by standard biochemical tests and the API 20E system (bioMérieux, Marcy l’Étoile, France) and serotyped by commercial antisera (Denka Seiken Co, Ltd., Tokyo, Japan). Antimicrobial drug susceptibility testing was performed with the standard Kirby-Bauer disk diffusion method by using commercially available antimicrobial disks (Becton Dickinson, Franklin Lakes, NJ, USA). Antimicrobial drugs tested for susceptibility were ampicillin, azithromycin (AZM), cefotaxime (CTX), ceftazidime (CAZ), ceftriaxone (CRO), ciprofloxacin (CIP), nalidixic acid (NAL), tetracycline, and trimethoprim/sulfamethoxazole. Susceptibility results were interpreted according to Clinical and Laboratory Standards Institute guidelines (*3*). We used zone diameter interpretive standards for *Enterobacteriaceae* for all antimicrobial drugs tested, except AZM, for which we applied the standard for *Staphylococcus* spp. 

*Shigella* spp. were isolated from 11 (5%) of 212 diarrhea stool samples. Antimicrobial drug susceptibility testing showed that 10 (91%) of the 11 *Shigella* isolates were resistant to ampicillin, tetracycline, trimethoprim/sulfamethoxazole, and NAL. We selected the 10 MDR isolates for further characterization and determined MICs of AZM and CIP by Etest (bioMérieux). ESBL production was tested by using Neg Combo Panel Type 50 on the MicroScan WalkAway plus System (Siemens Healthcare Diagnostics, Newark, DE, USA). PCR and sequencing were used to characterize resistance genes (*gyr*A and *par*C) in the quinolone-resistance determining region (QRDR), the AZM resistance gene (*mph*A), and β-lactamase genes (*4**–**7*).

Of the 10 MDR isolates, 2 were *S. flexneri* 2a; 1 was an *S. flexneri* 2 variant; 6 were *S. flexneri* 3a; and 1 was *S. sonnei* (Table 1). CIP resistance was detected in 5 (50%) of the 10 isolates. Sequence analysis showed mutations of *gyr*A and *par*C genes with the amino acid substitutions in the QRDR (Table 2). All NAL-resistant isolates susceptible to CIP had a single mutation in *gyr*A. Isolates resistant to both NAL and CIP contained multiple mutations in *gyr*A and *par*C.

**Table 1:** Epidemiologic data of patients with multidrug-resistant *Shigella*, Cambodia, July 2014–April 2015

Isolate no.	Organism	Isolate collection date	Patient age	Patient sex	Antimicrobial drugs taken before enrollment
1	*S. flexneri* 2a	2015 Apr 3	3 y	M	No
2	*S. flexneri* 2a	2015 Apr 28	18 mo	M	No
3	*S. flexneri* 2v	2015 Apr 20	1 y	F	No
4	*S. flexneri* 3a	2015 Jan 22	1 y	M	Yes*
5	*S. flexneri* 3a	2015 Feb 20	6 mo	M	No
6	*S. flexneri* 3a	2014 Nov 17	4 y	M	No
7	*S. flexneri* 3a	2014 Nov 25	1 y	F	Yes†
8	*S. flexneri* 3a	2014 Dec 12	3 y	M	No
9	*S. flexneri* 3a	2015 Feb 21	13 mo	M	No
10	*S. sonnei*	2015 Mar 11	2 y 4 mo	M	No
*Unknown type of drug taken 1 time. †250 mg amoxicillin taken for 3 d.					

**Table 2:** Antimicrobial susceptibility results and molecular characterization of resistance genes of *Shigella* isolates collected from patients in Cambodia, July 2014–April 2015*

Isolate no.	Organism	Antimicrobial resistance	CIP MIC, µg/mL†	Amino acid substitutions in QRDR					AZM MIC, µg/mL‡	*mph*A gene	ESBL confirmatory test	β-lactamase genes
				*gyr*A			*par*C					
				Ser 83	Asp 87		Ser 57	Ser 80				
1	*S. flexneri* 2a	AMP-SXT-TET-NAL	0.25	Leu	–		–	–	2.00	Neg	Neg	TEM-1, OXA-1
2	*S. flexneri* 2a	AMP-SXT-TET-NAL	0.25	Leu	–		–	–	1.50	Neg	Neg	TEM-1, OXA-1
3	*S. flexneri* 2v	AMP-SXT-TET-NAL	0.19	Leu	–		–	–	1.50	Neg	Neg	TEM-1, OXA-1
4	*S. flexneri* 3a	AMP-SXT-TET-NAL	0.25	Leu	–		–	–	1.00	Neg	Neg	TEM-1
5	*S. flexneri* 3a	AMP-SXT-TET-NAL	0.19	Leu	–		–	–	1.50	Neg	Neg	TEM-1
6	*S. flexneri* 3a	AMP-SXT-TET-NAL-CIP	4.00	Leu	–		Arg	–	0.75	Neg	Neg	TEM-1
7	*S. flexneri* 3a	AMP-SXT-TET-NAL-CIP	4.00	Leu	–		Arg	–	1.00	Neg	Neg	TEM-1
8	*S. flexneri* 3a	AMP-SXT-TET-NAL-CIP	4.00	Leu	–		Arg	–	1.00	Neg	Neg	TEM-1
9	*S. flexneri* 3a	AMP-SXT-TET-NAL-CIP-AZM-CRO-CTX	6.00	Leu	–		Arg	–	32.00	Pos	Pos	TEM-1, CTX-M-27
10	*S. sonnei*	AMP-SXT-TET-NAL-CIP-CRO-CTX-CAZ	6.00	Leu	Gly		–	Ile	4.00	Neg	Pos	CTX-M-55

*AMP, ampicillin; Arg, arginine; Asp, aspartate; AZM, azithromycin; CAZ, ceftazidime; CIP; ciprofloxacin; CRO, ceftriaxone; CTX, cefotaxime; ESBL, extended-spectrum β-lactamase; Gly, glycine; Ile, isoleucine; Leu, leucine; NAL, nalidixic acid; Neg, negative; Pos, positive; QRDR, quinolone-resistance determining region; Ser, serine; SXT, trimethoprim/sulfamethoxazole; TET, tetracycline; –, no amino acid substitutions found. †CIP MIC interpretive criteria for *Enterobacteriaceae* is susceptible <1, resistant >4 µg/mL. ‡AZM MIC interpretive criteria for *Salmonella enterica* serovar Typhi is susceptible <16, resistant >32 µg/mL.

The most common mechanism of quinolone resistance in the *Shigella* spp. was mutation of *gyr*A, typically at codon 83 or 87, and of *par*C at codon 80 (*7*). All isolates in our study had the common mutation in *gyr*A at position 83 (Ser83→Leu); 1 isolate had another common mutation at position 87 (Asp87→Gly). A mutation in *par*C at position 80 (Ser80→Ile), detected in the *S. sonnei* isolate, was previously reported in an *S*. *dysenteriae* serotype 1 isolate in India (*7*) and in Asia travel-associated *S. sonnei* and *S. flexneri* isolates in the United States (*8*). A mutation at position 57 (Ser57→Arg) was detected in all 4 CIP-resistant *S. flexneri* 3a isolates, but this mutation’s role in CIP resistance is unclear because position 57 is outside the QRDR region. Characterization of plasmid-mediated quinolone resistance (PMQR) genes should be further investigated because coexistence of mutations in the QRDR and PMQR genes has been reported in *Shigella* isolates with decreased susceptibility to fluoroquinolones (*8*). PMQR may facilitate the selection of QRDR mutations, resulting in higher levels of quinolone resistance.

No clinical breakpoints for AZM have been clearly defined for *Shigella* spp., but CDC’s National Antimicrobial Resistance Monitoring System for Enteric Bacteria (http://www.cdc.gov/narms/index.html) recommends using the term “decreased susceptibility” for reporting. We detected decreased susceptibility to AZM in *S. flexneri* 3a (isolate no. 9) with a MIC of 32 µg/mL. This isolate was found to carry the *mph*A gene encoding a macrolide 2′-phosphotransferase that inactivates macrolide antimicrobial drugs and has been reported to reduce AZM susceptibility in *Shigella* isolates (*5*). Emergence of decreased susceptibility to AZM may affect treatment options for shigellosis, especially for pediatric cases because ceftriaxone is administered parenterally by injection and fluoroquinolones are not encouraged for use in children.

We detected >1 β-lactamase gene in all 10 *Shigella* isolates; 2 isolates that were resistant to cephalosporins revealed ESBL production (Table 2). The 8 isolates that carried β-lactamase–producing genes TEM-1 or TEM-1 and OXA-1 were cephalosporin susceptible, suggesting that TEM-1 and OXA-1 may not play a role in increased resistance to third-generation cephalosporins. Of the remaining 2 isolates, 1 *S. flexneri* (isolate no. 9), which harbored CTX-M-27 and TEM-1, showed resistance to CRO and CTX but not to CAZ, and 1 *S. sonnei* (isolate no. 10), which carried CTX-M-55, was resistant to all cephalosporins tested.

A key element that increased ceftazidimase activity was a single amino acid substitution from Asp to Gly at position 240; this substitution was identified in CTX-M-15, CTX-M-16, CTX-M-27, and CTX-M-32. CTX-M-27, first reported from France in 2003, differed from its parental enzyme, CTX-M-14, by substitution of Asp240Gly (*9*). Reports suggest that this Gly-240–harboring CTX-M-27 confers higher levels of resistance to CAZ in *Escherichia coli* infections, but we did not detect this characteristic in the *Shigella* isolates we examined. CTX-M-55 was first reported in ESBL-producing *E. coli* and *Klebsiella pneumoniae* isolates in Thailand in 2007; it was associated with high resistance to CRO, CTX, and CAZ (*10*) and was subsequently reported in other Asia countries, including Cambodia. Among fecal samples collected from children in Cambodia, 88% carried *E. coli* harboring ESBL genes containing *bla*_CTX-M_ variants, including CTX-M-15, CTX-M-55, and CTX-M-14 (*11*). A case of ESBL-producing *S. sonnei* harboring CTX-M-55 was also reported in a woman traveling from Korea to China (*12*).

The CDC Health Alert Network has distributed a health advisory on CIP- and AZM-nonsusceptible *Shigella* infection in the United States (*13*). Three separate outbreaks of MDR shigellosis among men who have sex with men, international travelers, and children in daycare centers have been reported (*13*). We found 2 ESBL-producing, fluoroquinolone-resistant *Shigella* isolates. Moreover, *S. flexneri* 3a (isolate no. 9), which had decreased susceptibility to AZM, was also resistant to nearly all oral and parenteral drugs considered for shigellosis treatment. This isolate can ferment sorbitol, a feature found in 7% of *Shigella* spp. and possibly causing misidentification of *Shigella* spp. as other species (*14*). *S. sonnei* (isolate no. 10) belongs to biotype g (i.e., with biochemical reactions ONPG+ [o-nitrophenyl-β-D-galactopyranose], rhamnose–, and xylose–), which has been shown to carry integrons with multiple gene cassettes, leading to multidrug resistance (*15*).

## Conclusions

MDR *Shigella* is an emerging problem that raises concern about shigellosis treatment worldwide, including in Cambodia. Health authorities should implement systematic surveillance of antimicrobial drug resistance and controlled antimicrobial drug use to increase understanding of the problem and minimize unnecessary antimicrobial drug use, which contributes to increased resistance.
